# UAV path planning method for data collection of fixed-point equipment in complex forest environment

**DOI:** 10.3389/fnbot.2022.1105177

**Published:** 2022-12-22

**Authors:** Xiaohui Cui, Yu Wang, Shijie Yang, Hanzhang Liu, Chao Mou

**Affiliations:** ^1^School of Information Science and Technology, Beijing Forestry University, Beijing, China; ^2^Engineering Research Center for Forestry-Oriented Intelligent Information Processing, National Forestry and Grassland Administration, Beijing, China

**Keywords:** UAV, fixed-point path planning, multi-point path planning, two-point path planning, co-evolutionary algorithm

## Abstract

In a complicated forest environment, it is usual to install many ground-fixed devices, and patrol personnel periodically collects data from the device to detect forest pests and valuable wild animals. Unlike human patrols, UAV (Unmanned Aerial Vehicles) may collect data from ground-based devices. The existing UAV path planning method for fixed-point devices is usually acceptable for simple UAV flight scenes. However, it is unsuitable for forest patrol. Meanwhile, when collecting data, the UAV should consider the timeliness of the collected data. The paper proposes two-point path planning and multi-point path planning methods to maximize the amount of fresh information collected from ground-fixed devices in a complicated forest environment. Firstly, we adopt chaotic initialization and co-evolutionary algorithmto solve the two-point path planning issue considering all significant UAV performance and environmental factors. Then, a UAV path planning method based on simulated annealing is proposed for the multi-point path planning issue. In the experiment, the paper uses benchmark functions to choose an appropriate parameter configuration for the proposed approach. On simulated simple and complicated maps, we evaluate the effectiveness of the proposed method compared to the existing pathplanning strategies. The results reveal that the proposed ways can effectively produce a UAV patrol path with higher information freshness in fewer iterations and at a lower computing cost, suggesting the practical value of the proposed approach.

## 1 Introduction

Many ground-based fixed-point Internet of Things devices (also called IoT equipment) must be deployed during forest pest and disease prevention and rare species monitoring in order to collect ecological factors, various types of images, and other crucial data. Due to the fact that these devices are typically used in forests, which have high crown densities, complex environments, and no internet access, manual methods are typically used to regularly patrol fixed-point equipment in the forest and collect perception data. Manual data collection during patrols is time-consuming, ineffective, and increases patrol costs. Perceived data is frequently not obtained in a timely manner, leading to data quality issues like data loss. This is especially true when fixed-point equipment is deployed in challenging mountain environments. These have a significant impact on the precision of disaster and other warnings, as well as forest protection.

The UAV (unmanned aerial vehicle) has a wide load platform, high cruising efficiency on fixed routes, and other features. The data collected by the ground fixed-point equipment in the fixed-point cruise mode can be obtained in complex mountain environments by the multi-source sensor equipment mounted on the UAV, and the data can then be stored in the data center for further processing and analysis when the UAV returns. UAV patrol has gradually taken the place of manual patrol in maintaining some national forest parks and keeping an eye on the area close to cities.

Path planning allows for the prompt collection of data from fixed-point equipment deployed in the challenging mountain environment, solving the issue of manual data collection. Under a variety of constraints, including the performance of the UAV, its maximum flight length, potential environmental threats, flight altitude, maximum turning angle, and maximum climb/dive angle, traditional UAV path planning can be seen as a feasible flight path from the starting point to the target point. Numerous researchers, including (Yingkun, 2018; Hu et al., 2020; Kiani et al., 2022), have investigated UAV cruise problems and track path planning techniques and proposed a range of models and methods for urban security, agricultural patrol, and other scenarios. These techniques, however, typically only work for the starting and stopping points of a fixed-point track problem. The requirements of the forest point patrol data collection are not met because the scenario where the navigation needs to cover multiple points is not taken into account. The accuracy and timeliness of the data used in wildlife monitoring, forest disaster warning, and other specialized fields are also based on how quickly the patrol collects data. As a result, when the UAV is on patrol, the timeliness of data collection should also be prioritized.

Therefore, this paper focuses on the optimal trajectory planning problem of UAV fixed-point data collection in complex forest environments, with the goal of effectively obtaining a feasible trajectory planning path that satisfies fundamental requirements like flight performance and equal-height obstacle avoidance through the co-evolution method with domain adaptation. The perception data from all types of ground fixed point equipment has a significant time sensitivity when it comes to data collection during the forest patrol process. To assess how timely UAV data collection is, the age of information (AoI) index was created. As a result of AoI’s critical importance in patrol planning, AoI was included in the goals of UAV patrol planning. The paper’s specific contributions consist of:

(1) A path planning method for UAV fixed-point collection of forest floor monitoring data with comprehensive navigation performance, AoI, and other factors in complicated forest environments is provided. This method is targeted at time-sensitive forest monitoring tasks such as wildlife patrol, forest fire prevention, and pest monitoring.

(2) The plan took into account a wide range of variables, including the UAV data collection mode, the patrol or monitoring information timeliness, the UAV navigation posture, the obstacle avoidance in complex environments, and others. It methodically abstracted the objectives and constraints (terrain, threat, etc.) of UAV path planning in various intricate scenarios.

(3) An algorithm for co-evolving UAV patrol paths through complex forests is put forth. The algorithm continuously improves the dominant strategy applicable to the current scene from multiple evolutionary strategies through enhanced learning, then solves the UAV patrol flight path in an efficient and adaptive manner based on the characteristics of the patrol environment and AoI requirements.

## 2 Related work

Unmanned aerial vehicles path planning is a crucial component of UAV system design and serves as the foundation for UAV edge calculations, networking data transmission, and UAV flight control (Pehlivanoglu and Pehlivanoǧlu, 2021). At the moment, there are two main areas of UAV path planning research. The first step is to use domain problem modeling to attempt an accurate description of the domain characteristics and essential components of UAV solutions. The second is to design a UAV path planning algorithm that is efficient and satisfies the domain modeling characteristics.

Environment modeling and UAV modeling are two common examples of domain problem modeling. Environmental modeling is primarily used in UAV application scenarios, such as those involving city, mountain, and weapon threat models. Maximum flight height, maximum deflection angle, maximum pitch angle, maximum energy power, and longest flight path are among the flight characteristics that are primarily taken into account in UAV modeling.

Many researchers have adopted AoI as a crucial metric to assess the timeliness of data collection in UAV modeling in recent years because the perception data collected by UAV should be delivered to the data collector as soon as possible (Abbas et al., 2022). In 2012, (Kaul et al., 2012) first put forth the conception of AoI and described it as the interval from the time of packet generation to the present. Following that, many academics studied UAV path planning based on AoI and tailored to the needs of the field (Abd-Elmagid et al., 2019). Jointly optimized UAV flight trajectory using deep reinforcement learning framework, aiming for the least weighted sum of AoI (Jia et al., 2019) aiming to minimize the average AoI of sensors, optimizing UAVs’ flight trajectory and data collection mode through dynamic programming algorithms (Liu et al., 2018) took the maximum AoI optimal trajectory, averaged AoI optimal trajectory as targets, and used a dynamic programming algorithm and genetic algorithm to design the path planning of UAV in two-dimensional space (Tong et al., 2019) continued their research based on (Liu et al., 2018), using the nearest neighbor propagation algorithm to form sensor networks by clustering, reducing the influence of sensor size on algorithm efficiency through the specific collection points of each sensor network. Then designing the ideal trajectory using the dynamic programming algorithm (Liu et al., 2021) investigated the data collection technique used by unmanned aerial vehicles in wireless sensor networks based on AoI.

According to all of the aforementioned studies, AoI-based UAV path planning modeling has emerged as the most effective way to address the issue of the timing of domain perception data collection. The aforementioned studies, however, mainly concentrate on two-dimensional or approximate plane scenes. Complex forest scene patrol scenes cannot be directly applied to the relatively simple constraints taken into account in scene abstraction and the formation of target routes. Building a domain problem model for effective data collection from multi-point equipment at various altitudes based on AoI and complex conditions such as geographic factors, UAV performance, and UAV collision remains a necessary challenge for complex forest scene patrol.

In the aspect of UAV path planning algorithm design, heuristic algorithm or bionics algorithm has become the leading technical route. For example, Neural Networks (NNs) (Yan et al., 2020; Jin et al., 2022b; Shi et al., 2022; Xie et al., 2022), Dijkstra Algorithm (Maini and Sujit, 2016), Ant Colony Algorithm (ACO) (He et al., 2013), Artificial Potential Field Method (APF) (Chen et al., 2016a), Particle Swarm Optimization (PSO) (Zhang et al., 2013), Artificial Bee Colony (ABC) (Goel and Singh, 2013), Simulated Annealing Algorithm (SA) (Turker et al., 2015), and Genetic Algorithm (GA) (Xiao-Ting et al., 2013; Wang and Chen, 2014). Some scholars improved the above methods to build a more efficient UAV path-solving algorithm. Pehlivanoglu (2012) designed a multi-frequency vibration genetic algorithm (MVGA) that supports the Voronoi diagram to solve the problem of UAV path planning (Yang and Yoo, 2018) designed a UAV flight path planning method based on information such as no-fly zone, geographical positioning conditions, and sensor deployment by using a multi-objective biologically-inspired algorithm (Nikolos and Tsourvelouds, 2009) combined the traditional genetic algorithm and the improved proliferation genetic algorithm to design a path planner for the autonomous navigation of UAVs (Chen et al., 2016b) improved the path planning method through improved Particle Swarm Optimization (PSO) algorithm and Genetic Algorithm (GA) (Liu et al., 2016) solved the PSO algorithm’s efficiency problem in solving path planning from multiple perspectives, such as particle search space limitation and sensitivity.

The aforementioned research demonstrates that the key to increasing the effectiveness of UAV path planning is optimizing a single evolutionary strategy, coordinating multiple evolutionary strategies, and adapting to solve problems. However, in a complex forest environment, the UAV patrol path must deal with the accuracy of fixed point equipment’s data collection and ensure that UAV performance consumption and obstacle avoidance in a three-dimensional complex forest scene. The overall optimization algorithm structure, collaborative method of calculation strategy, algorithm execution efficiency, and domain problem ability must still be combined to create a UAV path planning algorithm with high problem execution efficiency, robustness, and scene generalization ability.

## 3 Description of problem

### 3.1 Scene modeling

**FIGURE 1:**
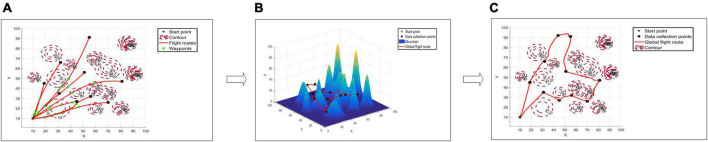
Unmanned aerial vehicles (UAV) forest fixed-points patrol diagram. Panel **(A)** is the top view of two-point path planning, panel **(B)** is the route map of multi-point path planning, and panel **(C)** is the top view of route map of multi-point path planning.

The actual patrol path of UAVs in a complex forest environment can be planned in two steps to gather data on ground equipment dispersed in various locations (Figure 1). In the first stage, a smooth multi-track flight path was built between the data collection points of two fixed points on the ground by combining flight obstacle avoidance, flight energy consumption, and other factors. The optimal path between every two points is successively obtained in the second stage, and the patrol path—capable of patrolling all the ground equipment at the points—is constructed on the basis of the smooth path between the points constructed in the first stage. The first stage, which will be abstracted in Section “3.1.1 Scenario of UAV two-point path planning,” can be represented as a flight path planning scenario of the flight path between two fixed-point devices. The second stage, which will be abstracted in Section “3.1.2 Scenario of UAV multi-point path planning,” can be represented as a global path planning scenario of flight paths between all fixed point equipment.

#### 3.1.1 Scenario of UAV two-point path planning

To ensure that the UAV can successfully avoid flight obstacles while adhering to energy consumption restrictions, take into account the complex landforms, numerous obstacles, and other features present in the forest scene that the UAV is inspecting. Thus, in order to create the three-dimensional path planning scene for the UAV, flight paths must be constructed for every pair of two points in the multi-point path planning in Section “3.1.2 Scenario of UAV multi-point path planning.”

##### 3.1.1.1 Form of solution in two-point programming scenario

**FIGURE 2:**
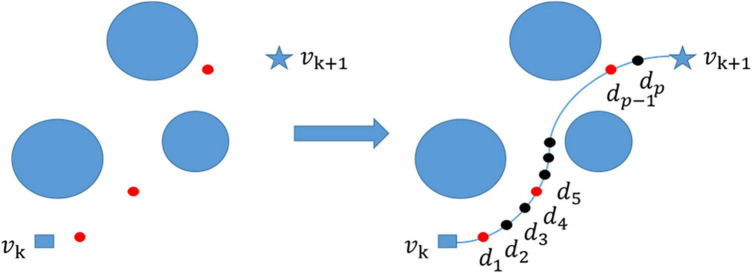
Path smoothing, generate a number of waypoints (red points) between the start point and end point, then generate a large number of intermediate points (black points) based on path smoothing, and finally connect them into a smooth curve.

In the two-point path planning, when there are obstacles such as high mountains in the process of flying from point v_k_ to point v_k + 1_, the flight path of the UAV is no longer a simple straight line, but connects point v_k_ to point v_k+ 1_ and avoids multiple obstacles. The three-dimensional spatial curve path (v_k_, d_1_, d_2_, …, d_p_, v_k + 1_) is composed of p intermediate points (Yang et al., 2015) (d_1_, d_2_, …, d_p_) (Figure 2).

##### 3.1.1.2 Two-point flight time representation

According to the two-point path planning scenario representation method, the time required for the UAV to move from point v_k_ and point v_k+ 1_ is shown in formula (1). η_(k),(k+ 1)_ represents the time spent going from v_k_ to v_k+ 1_ on the two-point path.


(1) $$\begin{array}{c} \eta_{\left(k\right),\left(k+1\right)}=\sum_{s=1}^{p+1}\frac{l_{s}}{v} \end{array}$$

Among this v represents the speed of the drone, and when s is 1, l_1_ represents the Euclidean distance from v_k_ to d_1_, l_s_ represents the Euclidean distance from d_s−1_ to d_s_,when s is p+1, l_p+ 1_ represents the Euclidean distance from d_p_ to v_k+ 1_.

#### 3.1.2 Scenario of UAV multi-point path planning

In the UAV multi-point path planning scenario, the UAV will fly over the ground sequentially to gather data on wildlife, forest and grass disaster warning, and other time-sensitive data collected at the fixed points (Liu et al., 2018). Each location is traversed by the UAV, which collects all the data and sends it back to the data center. In order to obtain and excavate crucial data in the forest and grass field, such as forest and grass early warning and priceless animal habitat, the data center will conduct additional analysis and processing of the data collected by the UAV.

##### 3.1.2.1 Form of solution in multi-point planning scenario

**FIGURE 3:**
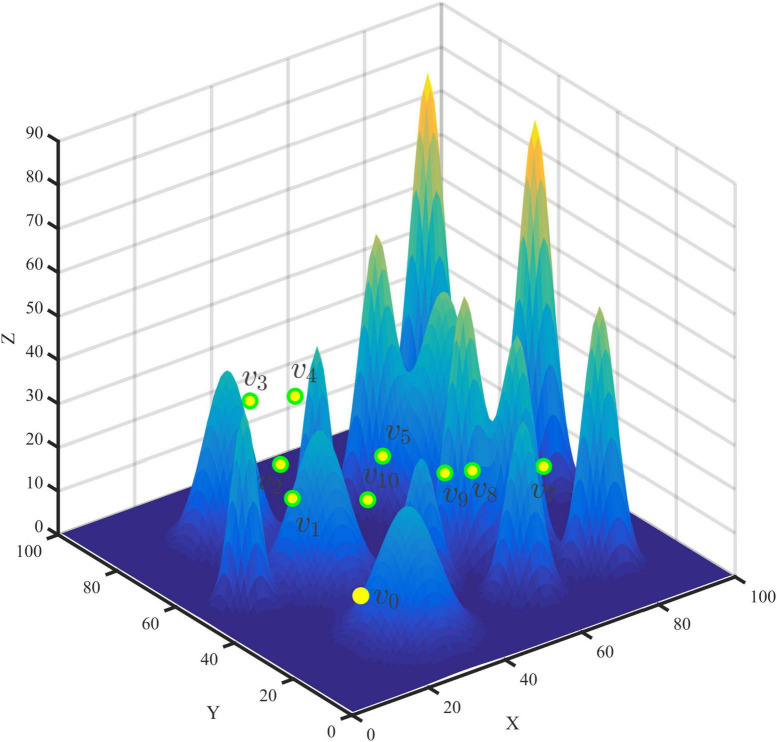
Multi-point diagram.

The set *V* = {v_1_,…,v_N_} is used to represent the location of fixed equipment to be flown over during UAV patrol, and the data center point of the UAV return position is indicated by v_0_. For any point v_k_ ∈ {v_0_} ∪V,v_k_ = (x_k_, y_k_, z_k_), among them, x_k_, y_k_, z_k_ represent the spatial location of the k-th data collection point, respectively, in multi-point path planning scenarios, this parameter must be met:


$$\{\left(x_{k}\cdot y_{k}\cdot z_{k}\right)|x_{m\,i\,n}\leq x_{k}\leq x_{m\,a\,x},$$

(2) $$y_{m\,i\,n}\leq y_{k}\leq y_{m\,a\,x},z_{m\,i\,n}\leq z_{k}\leq z_{m\,a\,x}\}$$

x_min_,x_max_,y_min_,y_max_,z_min_,z_max_ represent the boundaries of the environment, respectively. The solution of multi-point path planning can be regarded as taking v_0_ as the starting point and the endpoint, a primary loop containing each point in V, the node sequence with no repetition in the loop except v_0_, and each point in V appears, such as v_0_→v_1_ → v_2_ →—v_N_→v_0_,v_k_ ∈ V represents the k -th data collection point, and the form of the actual multi-point planning path in the UAV patrol scenario is shown in Figure 3.

##### 3.1.2.2 A representation of the age of information collected by a fixed-point device in a scenario

Since the data collected by UAV during patrol is time-sensitive, it is necessary to introduce the age of information and construct the representation method of the age of information. When the data is collected from the fixed-point device v_k_ and is collected when the UAV is hovering above v_k_ at t_k_, the age Γ_k_(t) of the collected data information in v_k_ can be represented as Γ_k_(t) = ^(t−t_k_) +^, where t_k_ represents the moment when the UAV reaches the k-th sensor node, (x) + max (Hu et al., 2020). ζ_*k*_ represents the time taken for *v*_*k*_ to upload the data to the drone. In multi-point path planning scenarios, it is generally assumed that the upload time of fixed point equipment, the flight time of UAV, the sampling time of each fixed point equipment and communication overhead can be ignored (Tong et al., 2019). The k-th node data information age is the sum of the upload data time on the path and the flight time of the UAV, as shown in formula (3).


(3) $$\begin{array}{c} \Gamma_{k}\,\left(t\right)=\sum_{i=k+1}^{n}\zeta_{i}+\sum_{i=k}^{n}\eta_{\left(i\right),\left(i+1\right)} \end{array}$$

Among these, η_(n),(n + 1)_ represents the time when the drone flies back to the data center from the last data collection point. According to formula (3), on the multi-point path planning, the average AoI (Tong et al., 2019) of the UAV flying over all ground fixed-point locations can be shown in formula (4).


(4) $$\begin{array}{c} \bar{\Gamma \,\left(t\right)}=\frac{1}{n}\,\sum_{k=1}^{n}\Gamma_{k}\,\left(t\right)=\frac{1}{n}\,\sum_{k=1}^{n}\left(\sum_{i=k+1}^{n}\zeta_{i}+\sum_{i=k}^{n}\eta_{\left(i\right),\left(i+1\right)}\right) \end{array}$$

### 3.2 Objective function

Two-point path planning and multi-point path planning make up the UAV forest patrol’s path planning, according to Section “3.1 Scene modeling.” The two scenarios mentioned above are combined to create the respective objective functions.

#### 3.2.1 Objective function of UAV two-point path planning

The objective function of multi-point path planning was built from the perspective of path length cost and mountain obstacle threat cost, taking into account the complex characteristics of the forest flight environment and the three-dimensional curve characteristics of the two-point patrol trajectory.

##### 3.2.1.1 Path length cost

Formula (1) states that the shorter the actual route length of the UAV’s two-point curve is, the less time is required, and the simpler it is to gather the perception data in a timely manner during obstacle avoidance flight. Formula (5) shows the cost of building the two-point path planning length.


$$m\,i\,n\,L=\sum_{s=1}^{p+1}l_{s}=\sum_{s=1}^{p+1}$$

(5) $$\sqrt{{\left(x_{s}-x_{s-1}\right)}^{2}+{\left(y_{s}-y_{s-1}\right)}^{2}+{\left(z_{s}-z_{s-1}\right)}^{2}}$$

Among this L represents the sum of distances between all adjacent intermediate track points from the two data collection points. p represents the number of intermediate points (x_s_,y_s_,z_s_) is the coordinate of the s-th intermediate node in the path, with s equal to 0, the starting coordinate v_k_, and s equal to p+1, the ending coordinate v_k+ 1_.

##### 3.2.1.2 Mountain obstacle threat cost

The primary obstacle avoidance targets for UAV forest patrol in complex forest environments are mountain peaks. Mountain obstacle threat costs are added to ensure that UAV can reasonably avoid mountain peaks, as shown in formula (6). Formula (6) states that the adaptability of this path can be decreased if the middle track point of two-point planning intersects with the surface of a mountain peak or is inside a mountain peak, leading to this path being viewed as an infeasible path in the planning process.


(6) $$\{\begin{array}{c} L=L*1000\;I\,f\,t\,h\,e\,c\,o\,l\,l\,i\,s\,i\,o\,n\\ L=L\;e\,l\,s\,e \end{array}$$

##### 3.2.2 Objective function of UAV multi-point path planning

To quantify the freshness of the data collected from UAV missions, many scholars have used AoI as an objective function for UAV trajectory optimization (Meng et al., 2021; Wu et al., 2021). In the process of forest patrol, to timely collect more valuable ground fixed point equipment perception data, the goal of UAV multi-point path planning is to minimize the average AoI of UAV data collection, that is, the minimum $\bar{\Gamma \,\left(\text{t}\right)}$.

### 3.3 Condition of constraint

The energy consumption, flight attitude, and other performance constraints as well as the environmental constraints of various UAVs must be taken into account when planning UAV tracks.

#### 3.3.1 Performance constraints

##### 3.3.1.1 Energy consumption constraints

The energy consumption model of UAV patrol is created based on the current mainstream fixed-wing and rotary-wing UAV products to guarantee that the energy consumed by UAV during flight must be less than its maximum energy.

Energy consumption model of rotary wing UAV. When the UAV flies at the speed v, the generated propulsion power consumption can be expressed as (Zeng et al., 2019):


(7) $$\begin{array}{c} P\,\left(v\right)=P_{0}\,\left(1+\frac{3\,v^{2}}{U_{t\,i\,p}^{2}}\right)+P_{i}\,{\left(\sqrt{1+\frac{v^{4}}{4\,v_{0}^{4}}}-\frac{v^{2}}{2\,v_{0}^{2}}\right)}^{\frac{1}{2}}+\frac{1}{2}\,d_{0}\,\rho \,s\,A\,v^{3} \end{array}$$

In formula (7), ${\text{P}}_{0}\,\left(1+\frac{3\,{\text{v}}^{2}}{{\text{U}}_{\text{tip}}^{2}}\right)$ is leaf profile power, ${\text{P}}_{\text{i}}\,{\left(\sqrt{1+\frac{{\text{v}}^{4}}{4\,{\text{v}}_{0}^{4}}}-\frac{{\text{v}}^{2}}{2\,{\text{v}}_{0}^{2}}\right)}^{1/2}$ is induced power, and $\frac{1}{2}\,{\text{d}}_{0}\,\rho \,{\text{sAv}}^{3}$ is parasitic power. Among them, P_0_ and P_i_ is constant, respectively for the UAV hovering state blade profile power and induced power, U_tip_ said rotor blade tip speed, ν_0_ means hovering rotor average induction speed, d_0_ and s, respectively for UAV fuselage resistance ratio and rotor strength, related to the type of UAV, ρ and A, respectively said air density and rotor disk area.

Energy consumption model of fixed-wing UAV. The energy consumption model of fixed-wing UAV depends on the flight speed and acceleration of the UAV (Hua et al., 2018), which can be expressed as


(8) $$\begin{array}{c} P\,\left(\nu ,\alpha \right)=c_{1}\,{\left|v\right|}^{3}+\frac{c_{2}}{||v||}\,\left[1+\frac{{||a||}^{2}-\frac{{\left(a^{T}\,v\right)}^{2}}{{||v||}^{2}}}{g^{2}}\right]+m\,a^{T}\,v \end{array}$$

Among these ν and α are the flight speed and acceleration of the UAV, respectively, c_1_ and c_2_ are the parameters related to its weight, wing area, and air density, *g* = 9.8m/s^2^ is the gravity acceleration, and m is the mass of the UAV and its load. When the fixed-wing UAV flies smoothly at speed v, its energy consumption model can be reduced to the following form


(9) $$P=c_{1}\,{||v||}^{3}+\frac{c_{2}}{||\nu ||}$$

The propulsion energy of a UAV during flight can be expressed as:


(10) $$\begin{array}{c} E_{F}\,\left(t\right)=\delta \,\left\lfloor c_{1}\,{||v||}^{3}+\frac{c_{2}}{||\nu ||}\right\rfloor \end{array}$$

The c_1_ and c_2_ are constant and are related to UAV weight, wing area, air density, etc. The δ is the length of time in the unit time slot.

##### 3.3.1.2 Flight attitude constraints

The maximum horizontal rotation angle, the maximum climbing angle, and the minimum and maximum flight altitude of the UAV flight will all have an impact on the flight energy consumption in the three-dimensional path flight.

###### 3.3.1.2.1 Maximum turning angle

First, the UAV’s turning angle should not be too large due to its own performance constraints. Second, while turning, the UAV will slightly veer from the intended flight path; at this point, the flight path curvature should be taken into account to maintain a safe distance between the flight path and the terrain. In other words, it should not exceed the maximum turning Angle.

###### 3.3.1.2.2 Maximum climbing angle

The ability of the UAV to maneuver, flight altitude, and weather conditions are the main factors limiting the climbing angle of the UAV in 3D space flight path planning. Therefore, during flight, the UAV’s climbing Angle should not exceed its maximum climbing Angle.

###### 3.3.1.2.2 Minimum and maximum flight altitude

The minimum altitude of each track point in the UAV track search must not be less than a specified terrain altitude, and the flight altitude must not be higher than the maximum flight altitude.

#### 3.3.2 Environmental constraints

The main danger to UAVs using ground sensors in challenging mountain environments is the danger posed by the mountains. UAVs should therefore avoid running into mountains while in the air.

### 3.4 Path smoothing

To ensure that the planned path of two points can be used as the smooth trajectory of actual UAV flight, it is also necessary to construct a smooth flight path for the track points between two adjacent points in multiple points. Based on this, cubic B-spline curve (Chai et al., 2022) was introduced in this paper to smooth the flight path of UAVs and build a feasible flight path. The red points represent the algorithm-generated waypoints and the black points represent the points generated by the cubic B-spline curve (Figure 2).

## 4 Materials and methods

### 4.1 Logistic chaos initialization

Chaos is a spontaneously generated instability in deterministic systems, commonly found in nonlinear systems. Due to the ergodic nature of chaos, cango through all states in a certain range without repetition. Using chaotic variables for optimization is superior to blind and disorderly random searches. In this paper, chaos theory is introduced to population initialization. The main idea of chaotic search is to generate chaotic sequences in some iterative way, and generally most logistic equations are used to generate chaotic sequences, as shown in Equation (11):


(11) $$\begin{array}{c} r_{i+1}=\mu \,r_{i}\,\left(1-r_{i}\right)\;i=1,2,\ldots ,n \end{array}$$

The logistic mapping is in a fully chaotic state at the bifurcation parameter 3.57< μ ≤ 4, and the trajectory of the equations in this interval shows chaotic characteristics. In this paper, we take μ = 3.98.

### 4.2 Coevolution algorithm

#### 4.2.1 The introduction of coevolution algorithm

No single algorithm is the most efficient for solving all optimization problems, according to the “No-free-lunch” theorem, and many researchers are working to increase the generalization ability of the approaches by hybridizing the characteristics of the problems that have been solved (Shi and Zhang, 2020; Jin et al., 2022a; Liu et al., 2022).

Coevolution algorithms have the ability to actively learn the properties of the solution space in accordance with the properties of the issue, adaptively select the dominant strategy suitable for the algorithm’s convergence from a variety of evolutionary strategies, significantly increase the algorithm’s generalization ability to the problem, and produce superior results while addressing active complex optimization problems.

In a complex forest environment, UAV two-point path planning is the key to avoiding obstacles and collecting fixed-point data efficiently. This paper uses GA and SA strategies to design the parallel evolutionary algorithm (GASA). Under parameter control, the GA is an adaptive global search strategy, and the SA can reflect good local search capabilities. During the co-evolutionary process, the two evolutionary techniques complement each other and can improve the algorithm’s convergence speed and improve path planning efficiency.

#### 4.2.2 Two-point path planning using the GASA coevolution algorithm

##### 4.2.2.1 Setting of algorithm parameters

Set the relevant parameters and map boundaries of the GASA coevolution algorithm, which include population size popNum, chromosome length chromLength, crossover probability p_crs, mutation probability p_mut, iteration number iterMax, initial temperature T, termination temperature Tf, annealing rate k, Markov chain length L, and probability selection step r.

##### 4.2.2.2 Logistic chaos initialization in two-point path planning

The set of waypoint coordinates between two data collection points is considered as an individual, the chromosome length of the individual is chromLength, and the path between data collection point *v_k* and data collection point *v*_*k+1*_ is denoted as (*v*_*i*_,*d*_1_,*d*_2_,…,*d*_*p*_,*v*_*i* + 1_). First take the random number vectors *a_1*, *b_1*, *c_1* initial values between 0 ∼ 1.


(12) $$a_{1}=\left(a_{1}^{1},a_{1}^{2},\ldots ,a_{1}^{c\,h\,r\,o\,m\,L\,e\,n\,g\,t\,h}\right)$$

(13) $$b_{1}=\left(b_{1}^{1},b_{1}^{2},\ldots ,b_{1}^{c\,h\,r\,o\,m\,L\,e\,n\,g\,t\,h}\right)$$

(14) $$c_{1}=\left(c_{1}^{1},c_{1}^{2},\ldots ,c_{1}^{c\,h\,r\,o\,m\,L\,e\,n\,g\,t\,h}\right)$$

Then a new vector is generated according to the form of the logistic mapping.


(15) $$a_{i+1}=\mu \,a_{i}\,\left(1-a_{i}\right)$$

(16) $$b_{i+1}=\mu \,b_{i}\,\left(1-b_{i}\right)$$

(17) $$c_{i+1}=\mu \,c_{i}\,\left(1-c_{i}\right)$$

Where *i* = 1,2,…,*popNum*

The resulting chaotic variables are mapped into the three-dimensional space to obtain the set of three-dimensional coordinates of the i-th individual of the initial population. The i-th individual in the population *pop*_*i*_ = (*X*_*i*_,*Y*_*i*_,*Z*_*i*_).


(18) $$\begin{array}{c} X_{i}=\left(x_{m\,a\,x}-x_{m\,i\,n}\right)*a_{i}+x_{m\,i\,n} \end{array}$$

(19) $$\begin{array}{c} Y_{i}=\left(y_{m\,a\,x}-y_{m\,i\,n}\right)*b_{i}+y_{m\,i\,n} \end{array}$$

(20) $$Z_{i}=\left(z_{m\,a\,x}-z_{m\,i\,n}\right)*c_{i}+z_{m\,i\,n}$$

##### 4.2.2.3 GA strategy

Superior genes can be kept in the population by using selection, crossover, and mutation operations to create new populations and keeping elite particles out of crossover and mutation operations.

##### 4.2.2.4 SA strategy

Randomly select an individual for annealing operation to generate a new population of the received particles. After the evolution is finished, the annealing temperature of the SA strategy is updated in time.

##### 4.2.2.5 Adjust the solution according to the constraint

Optimize populations by eliminating some particles according to constraints.

##### 4.2.2.6 Update strategy selection probability

After the evolution of the GA strategy or SA strategy, the parent population and the child population are fused and ranked according to the fitness value. The individuals with the population are retained to form a new population. Suppose the number of individuals from the parent population is more than the number of individuals from the offspring population. In that case, the corresponding algorithm is the dominant strategy and the probability of selection of this algorithm should be increased according to formula (21), and vice versa, the probability of selection of the algorithm should be decreased according to formula (22).


(21) u(l)=u(l)+r*(1-u(l)

(22) u⁢(l)=u⁢(l)-r*u⁢(l)

The pseudo-code for solving the shortest path between two points (v_i_,v_j_) using the GASA coevolution algorithm is shown in Table 1.

**TABLE 1:** GASA coevolution algorithm pseudo-code to solve the optimal path between two points(*v*_*i*_,*v*_*j*_).

Input:	The start point start point *v*_*i*_ and end point *v*_*j*_
Output:	An optimal path according to cubic *B*-spline curve
1	Initialize map parameters and set GASA algorithm parameters
2	Initialize population based on logistic Chaos
3	Evaluate population and keep global_best
4	for i from 1 to iterMax do
5	if a random number of 0–1 is less than the GA selection probability then
6	Select out superior individuals for crossover operations
7	Select some individuals for mutation operation
8	Optimize population
9	Evaluate population and update global_best
10	Converge populations and update strategy selection probability
11	else
12	Randomly select a particle *X*_0_
13	while *T* > Tf do
14	T = k*T
15	for m from 1 to L do
16	Generate a new individual *X*_new_ based on *X*_0_ and evaluate (*X*_new_)
17	if the result of *X*_new_ is better than global_best then
18	Update global_best and accept new particle *X*_new_
19	end if
20	if the result of *X*_new_ is better than the previous particle *X*_m_ then
21	Accept new particle *X*_new_
22	else
23	Calculate the probability *p* in the Metropolis criterion
24	if a random number of 0–1 is less than *p* then
25	Accept new particle *X*_new_
26	end if
27	end if
28	end for
29	end while
30	Converge populations and update strategy selection probability
31	end if
32	end while

### 4.3 Multi-point path planning using simulated annealing algorithm

A full alignment of the data collection points makes up one particle of the simulated annealing algorithm, and the process calculates the total fitness value of the entire path, accepting and updating the global optimum if it is better than the global one, accepting if it is better than the previous one, and accepting probabilistically if it is worse than the previous one.

The locations of the data collection points to be flown by the UAV in this study are known, and the UAV starts from a fixed starting point *v_0* and flies over each fixed point device only once, and finally returns to the starting point *v_0* with the minimum average AoI $\bar{\Gamma \,\left(t\right)}$of all nodes.

**FIGURE 4:**
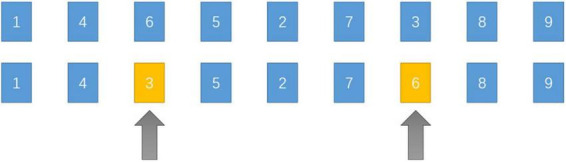
Swap operation.

It is known from formula (1) that the timeliness of the data and the flight path are proportional to one another when the UAV flies at a particular speed. This study applies the GASA coevolution algorithm to discover the shortest path between two points in order to achieve the time-optimal path of the UAV from the current collection point to the next target point. This work employs the simulated annealing process to determine the best solution for the issue of minimizing the average information age of all nodes; the algorithm pseudo-code is displayed in Table 2. Figure 4 depicts the SA algorithm swap operation.

**TABLE 2:** GASA coevolution algorithm + SA algorithm pseudo-code to solve the global optimal path.

Input:	Input *N* data collection points
Output:	An optimization path with minimum AoI
1	for i from 1 to N do
2	for j from i to N do
3	Calculate the optimal path of v_i_ to v_j_
4	end for
5	end for
6	Randomly select a global path *P* and evaluate P
7	while *T* > Tf do
8	for i from 1 to L do
9	Generate a new path *P*_new_ by swapping two points
10	if the AoI of *P*_new_ is smaller than *P*_best_
11	Update *P*_best_ and accept new path *P*_new_
12	end if
13	if the AoI of *P*_new_ is smaller than *P*
14	Accept new path *P*_new_
15	else
16	Calculate the probability *p* in the Metropolis criterion
17	if a random number of 0–1 is less than *p* then
18	Accept new path *P*_new_
19	end
20	end if
21	end for
22	end while

## 5 Experiment

### 5.1 Summary of experiment

Four distinct sorts of experiments are set in this chapter to test the usefulness of the suggested method. The first experiment is a fundamental experiment for selecting the proper probability choice step parameter r, the second experiment is an experiment to verify the algorithm on the Benchmark function, and the third experiment is an experiment to compare two-point path planning simulations based on simulation maps. The fourth experiment is a multi-point comparative simulation experiment involving multiple data gathering points in two different circumstances.

On a Windows 10 computer with an Intel Core i7-6700HQ processor, 8 GB of memory, and the Matlab2016a platform, all experiments in this paper were simulated.

### 5.2 Probability selection step parameter

Theoretically, selecting a greater *r* will cause the algorithm to be over-sensitive to changes in the probability of strategy selection, resulting in over-fitting. When *r* is too little, the sensitivity to a problem is insufficient to achieve the benefit of adaptive learning. To further determine the optimum probability selection step parameter *r*, this work analyzes the statistics of the adaptation of the matching results provided by GASA when *r* = 0.1, 0.2, 0.3, 0.4, 0.5, and presents the results of 3-dimensional experiments as an example. The values in Table 3 are the mean, median, and standard deviation of the Ackley function and Griewank function in 10 independent experiments.

**TABLE 3:** Function values obtained for the different *r*.

		*r* = 0.1	*r* = 0.2	*r* = 0.3	*r* = 0.4	*r* = 0.5
Ackley	Average	0.059356	**0.051123**	0.083617	0.064636	0.072232
	Median	0.063423	**0.049352**	0.067312	0.053876	0.072025
	Standard deviation	0.03689	**0.018303**	0.068941	0.041195	0.032264
Griewank	Average	0.01274	**0.00808**	0.00976	0.01122	0.00975
	Median	0.010217	**0.009962**	0.010012	0.009993	0.010957
	Standard deviation	0.007427	**0.004097**	0.003337	0.006903	0.004048

The bold values are the suitable ones.

According to the experimental results, the various statistics are better when *r* = 0.2. In the subsequent experiments, *r* = 0.2.

### 5.3 Benchmark function experiments

**FIGURE 5:**
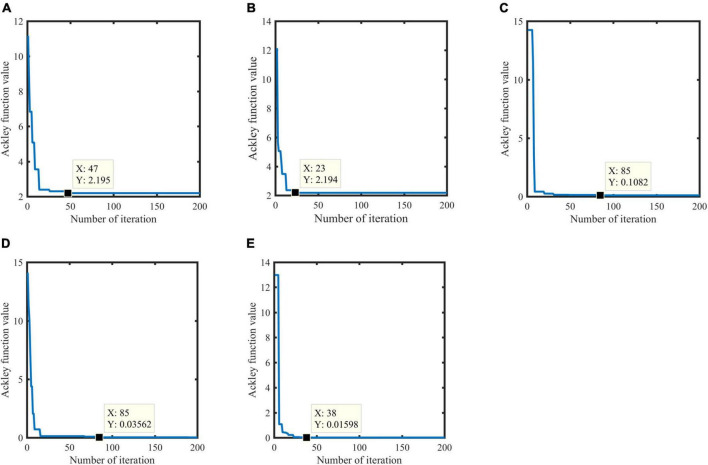
Results based on the Ackley function. Genetic Algorithm (GA) algorithm **(A)**, GA algorithm based on logistic chaos initialization **(B)**, Simulated Annealing Algorithm (SA) algorithm **(C)**, GASA coevolution algorithm **(D)**, an GASA coevolution algorithm based on logistic chaos initialization **(E)**.

In order to prove the effectiveness of chaotic initialization, this paper tests the convergence rate and optimal function value of GA algorithm, Logistic chaotic initialization-based GA algorithm, SA algorithm, GA algorithm, GASA coevolution algorithm, and Logistic chaotic initialization based GASA coevolution algorithm on Ackley function. The experimental parameters are shown in Table 4. The experimental results (Figure 5) show that GA algorithm converges in the 47th generation, and the optimal Ackley function value is 2.196. The GA algorithm improved based on logistic chaos initialization converges in the 23rd generation, and the optimal Ackley function value is 2.194, which can accelerate the convergence speed of GA algorithm. Although the two final results are similar, the convergence speed of the improved GA algorithm is increased by 100%. Logistic chaos initialization can accelerate the convergence of GA algorithm. Compared with the other three algorithms, the GASA coevolution algorithm based on logistic chaos initialization has better convergence speed and accuracy and optimization ability.

**TABLE 4:** Main parameters of GASA coevolution algorithm on the Benchmark function.

Parameter	Symbol	Parameter values
Upper limit	Xs	32
Lower limit	Xx	−32
Population size	popNum	300
Chromosome length	chromLength	3
Iteration number	iterMax	100
Selection probability	p_select	0.8
Crossover probability	p_crs	0.8
Mutation probability	p_mut	0.2
Initial temperature	*T*	10
Regulatory factor	*k*	0.95
Length of Markov chain	*L*	20
Final temperature	Tf	0.1
Initial selection probability	initialProbability	0.5
Probability selection step	*r*	0.2

### 5.4 Two-point path planning experiment

This paper constructs a simulation map for the simulation experiment to test the validity of the GASA coevolution algorithm in the path planning problem between two points. To test the effect of population size and the number of iterations on the GASA coevolution algorithm, we fine-tuned the selection range of parameters and did several sets of comparison experiments, and the results of the experiments showed that the effect of the parameters was small, and we later chose the most suitable parameters to compare with other algorithms. It contrasts the PSO algorithm, GA algorithm, ACO algorithm, and ABC algorithm in terms of convergence speed, path cost, and three-dimensional track diagram. Table 5 displays the precise experimental parameters.

**TABLE 5:** Main parameters of GASA coevolution algorithm in two-point path planning.

Parameter	Symbol	Parameter values
Execution space	–	100 × 100 × 100
Start point	startPos	[10,10,20]
End point	goalPos	[68,47,24]
Population size	popNum	50
Chromosome length	chromLength	3
Iteration number	iterMax	30
Selection probability	p_select	0.8
Crossover probability	p_crs	0.8
Mutation probability	p_mut	0.2
Initial temperature	*T*	10
Annealing rate	*k*	0.95
Markov chain length	*L*	20
Termination temperature	Tf	0.1
Initial selection probability	initialProbability	0.5
Probability selection step	*r*	0.2

The convergence speed and objective function values of all algorithms are shown in Table 6. GASA coevolution algorithm converges in the 5th generation. The optimal path length is 70.34, and the linear distance between the two points of the starting point and the end point is 68.91, which is close to that of the GASA coevolution algorithm. The path cost of the GASA coevolution algorithm is much lower than the GA and ACO algorithms. Although the final objective function value of PSO algorithm is not much different from that of the GASA coevolution algorithm, the PSO algorithm does not converge until the 60th generation, and the convergence speed is slower than that of the GASA coevolution algorithm. The optimal path length obtained by the ABC algorithm is 71.91, but it does not converge until the 70th generation, and the convergence speed is slower. It can be seen from the above that GASA coevolution algorithm has the optimal planning result, which can effectively restrain the precocity of traditional genetic algorithm and accelerate the rate of algorithm convergence.

**TABLE 6:** Comparison of every algorithm in two-point path planning.

Algorithm name	Two straight distance	Algorithm for the optimal path length	Convergence iteration number
GASA (popNum = 150, iterMax *= 30*)	68.91	70.74	19
GASA (popNum = 100, iterMax *= 30*)	68.91	70.50	25
GASA (popNum = 70, iterMax *= 50*)	68.91	70.54	21
GASA (popNum = 50, iterMax *= 30*)	**68.91**	**70.34**	**5**
GASA (popNum = 30, iterMax *= 20*)	68.91	70.74	20
PSO	68.91	74.14	60
GA	68.91	144.20	22
ACO	68.91	94.89	19
ABC	68.91	71.91	70

The bold values are the suitable ones.

**FIGURE 6:**
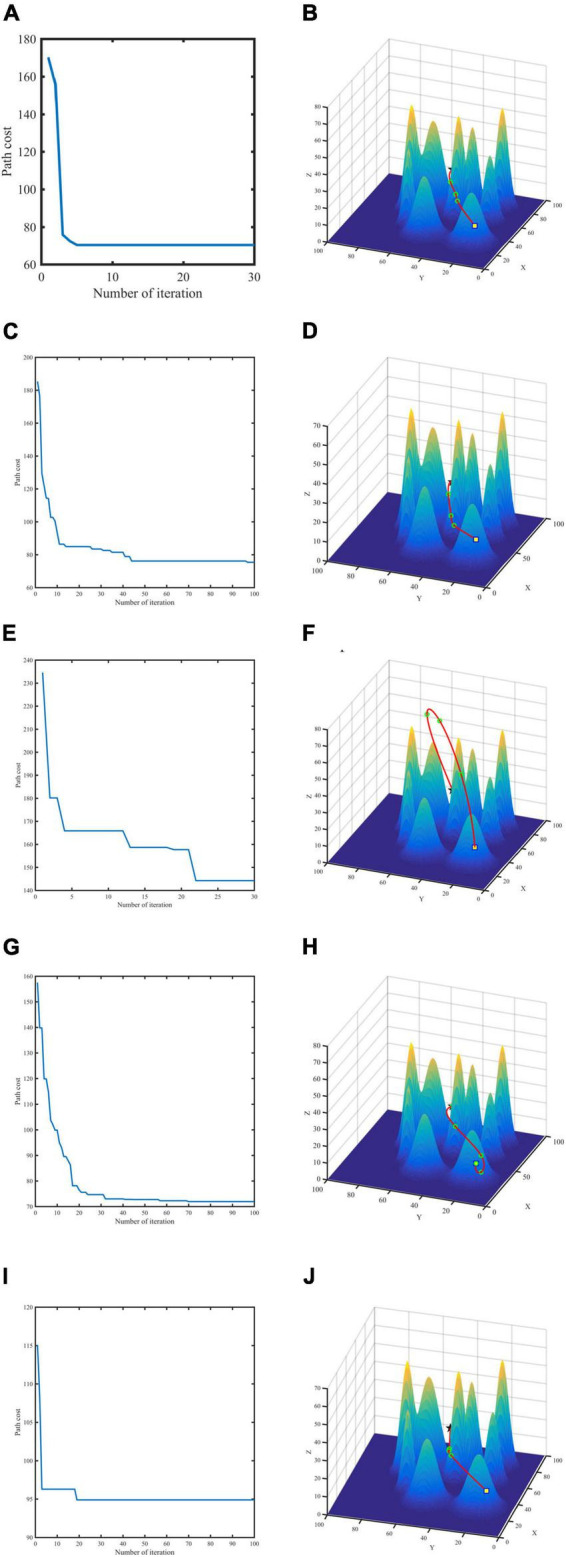
Convergence curve and flight path for two-point planning. GASA **(A,B)**, Particle Swarm Optimization (PSO) **(C,D)**, Genetic Algorithm (GA) **(E,F)**, Ant Colony Algorithm (ACO) **(G,H)**, Artificial Bee Colony (ABC) **(I,J)**.

From the experimental simulation results (Figure 6), it can be seen that the GASA coevolution algorithm proposed in this paper can plan the flight path of UAVs and improve the optimization accuracy of the original algorithm. Compared with the GA algorithm, GASA coevolution algorithm can better jump out of the local optimal in the middle and later optimization stage, restrain algorithm precocity and accelerate algorithm convergence speed. Moreover, compared with the GA algorithm, PSO algorithm, ACO algorithm and ABC algorithm, GASA coevolution algorithm has higher optimization accuracy and faster convergence speed. It can better plan an optimal flight path for UAVs.

### 5.5 Path planning experiment between multiple points

**FIGURE 7:**
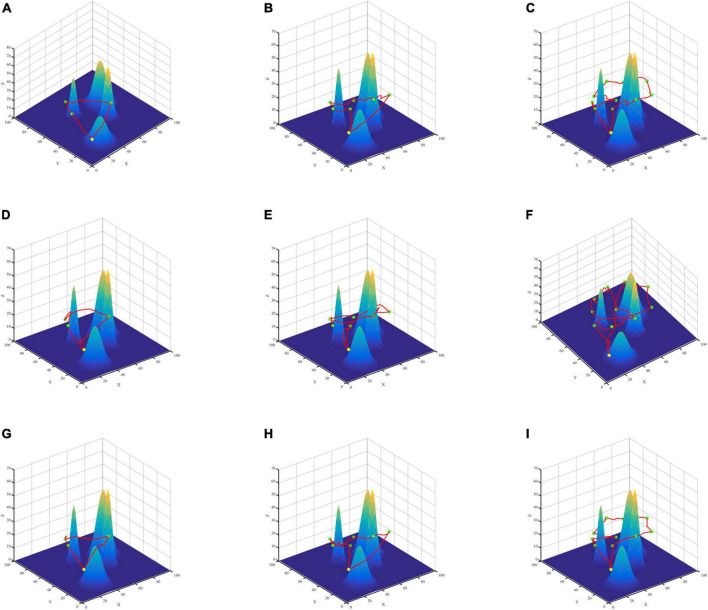
Three algorithms for multi-point path planning trajectory maps in simple maps. GASA **(A–C)**, Particle Swarm Optimization (PSO) **(D–F)**, Artificial Bee Colony (ABC) **(G–I)**.

**FIGURE 8:**
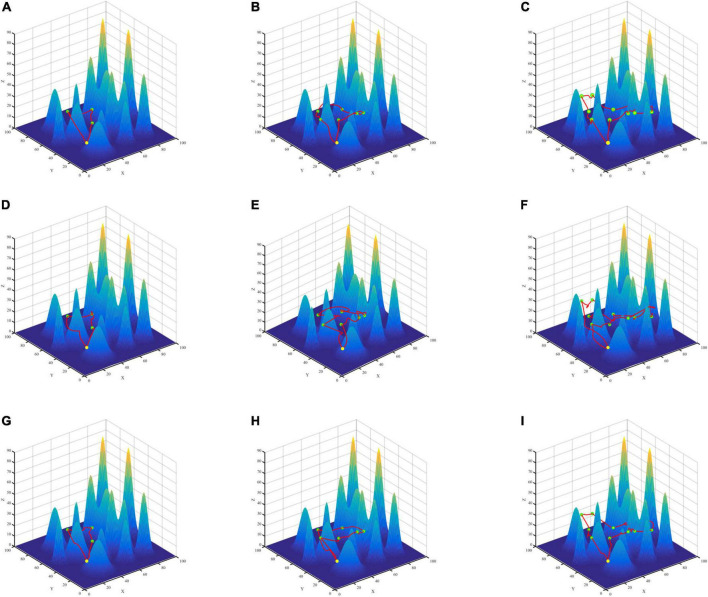
Three algorithms for multi-point path planning trajectory maps in complex maps. GASA **(A–C)**, Particle Swarm Optimization (PSO) **(D–F)**, Artificial Bee Colony (ABC) **(G–I)**.

To verify the effectiveness of the proposed algorithm in multi-point path planning, this paper constructs two different terraforms, simple scenario, and complex scenario (Figures 7, 8), sets different numbers of data collection points, and compares the average AoI of data collection points. The starting point of the route is (10,10,20). We employ the PSO algorithm for comparison to test the validity of SA for multiple-point planning. In addition, for the inner two-point path planning algorithm, we compare the PSO and ABC algorithms from Section “5.4 Two-point path planning experiment” with the GASA collaborative algorithm. The experimental results indicate that the SA algorithm is a highly stable method. When conducting path planning experiments in simple and complicated maps, the average AoI of SA algorithm is better than that of the PSO algorithm, according to Table 7.

**TABLE 7:** Average information age (AoI) comparison table of the different maps of every algorithm.

		Simple scenario			Complex scenario		
Outer layer algorithm	Inner layer algorithm	Three data collection points	Six data collection points	10 data collection points	Three data collection points	Six data collection points	10 data collection points
SA	Average AoI of GASA algorithm	20.7315	56.2689	150.1866	19.12	54.75	140.79
	Average AoI of PSO algorithm	24.7728	70.9382	195.9088	21.5919	67.9913	187.3861
	Average AoI of ABC algorithm	21.854	58.5331	152.451	19.4519	55.035	141.1853
PSO	Average AoI of GASA algorithm	20.7315	56.2689	147.125	19.12	54.75	144.158
	Average AoI of PSO algorithm	24.7728	70.9382	185.6892	21.5919	67.9913	194.7268
	Average AoI of ABC algorithm	21.854	58.5331	187.1666	19.4519	53.035	147.8047

According to the results (Table 7), compared with PSO algorithm and ABC algorithm, the GASA algorithm proposed in this paper can reduce the average AoI of the collected data and improve the freshness of the data. Compared with the PSO algorithm, the average AoI of data in the simple map is reduced by 16.31, 20.67, and 23.33% at 3, 6, and 10 data collection points, respectively. In the complex map, the average data AoI decreased by 11.45, 19.47, and 24.87% at 3, 6, and 10 data collection points, respectively. Compared with the ABC algorithm, the average AoI of data in the simple map is reduced by 5.14, 3.87, and 1.49% at 3, 6, and 10 data collection points, respectively. In the complex map, the mean AoI of data decreased by 1.7, 0.05, and 0.02% at 3, 6, and 10 data collection points, respectively.

## 6 Conclusion

In this paper, the UAV path planning method in complex environment is designed for forest pest monitoring, wildlife protection and resource monitoring process, where the UAV needs to efficiently collect data from fixed-point monitoring devices. With the information freshness as the main optimization objective, the two-point and multi-point path planning process is integrated to design the UAV path planning method facing the complex mountain environment. In the multi-point path planning, the UAV patrol path between multiple fixed-point devices is obtained by simulated annealing method with the goal of information freshness. In the two-point path planning, the UAV 3D smooth patrol path between two fixed-point devices is obtained by integrating information freshness, flight attitude, and complex environment elements through the coevolution algorithm. In the experiment, the reasonable configuration of parameters of the proposed method is determined using the benchmark function, and then, the effectiveness of the two-point path planning method and the multi-point path planning method is verified using simulated complex mountain environments with different configurations of the number of fixed points. Then, the effectiveness of the two-point path planning method and the multi-point path planning method is verified using simulated complex mountain environments with different configurations of the number of fixed points. When the outer algorithm uses the SA algorithm and the inner two-point path planning GASA coevolution algorithm is compared with the existing method PSO, the proposed two-point optimization method reduces 11.4, 19.4, 24.8% in terms of AoI in the complex environment, and when the inner algorithm When the inner layer algorithms are the same, the proposed multi-point optimization method SA is more stable in the complex environment, and the AoI is reduced by 2.3, 3.7, and 4.5%.

The method proposed in this paper is an exploration of the UAV with fixed time path planning method for complex mountainous environments. In the subsequent research, the applicability and practical application value of the algorithm can be improved in terms of scene migration and method improvement. In the scenario migration, the multi-objective optimization method applicable to other UAV patrol scenarios can be constructed by combining the UAV patrol needs of simple urban suburban environments and introducing airtime, etc. as the objective function. In the convenience of method improvement, it can be combined with the actual wildlife monitoring needs and domain expert knowledge to construct the illuminating information optimization objective function of the optimization method, meanwhile, some new optimization methods can be combined with the existing methods to construct the co-evolutionary algorithm to verify the applicability of different evolutionary strategies in the forest patrol problem.

## Data availability statement

The raw data supporting the conclusions of this article will be made available by the authors, without undue reservation.

## Author contributions

XC and CM: conceptualization, writing—review and editing, and funding acquisition. XC, YW, and SY: methodology. YW and HL: coding. XC and YW: writing—original draft preparation. XC, YW, and CM: significance contributions to the manuscript. All authors read and agreed to the published version of the manuscript.
